# Pancreatic cancer: genetics, disease progression, therapeutic resistance and treatment strategies

**DOI:** 10.20517/2394-4722.2021.96

**Published:** 2021-11-05

**Authors:** Karnika Singh, Gauri Shishodia, Hari K. Koul

**Affiliations:** 1 Department of Radiation Oncology, The Ohio State University Comprehensive Cancer Center, Columbus, OH 43210, USA.; 2 Department of Otolaryngology/Head & Neck Cancer Surgery, LSU Health Sciences Center, Shreveport, LA 71103, USA.; 3 Department of Biochemistry & Molecular Biology, Urology and Stanley S Scott Cancer Center School of Medicine LSU Health Sciences Center, New Orleans, LA 70112, USA.

**Keywords:** Pancreatic cancer, signature mutations, gemcitabine, desmoplasia, therapeutic resistance, immunotherapy

## Abstract

Pancreatic cancer is a deadly disease and the third-highest cause of cancer-related deaths in the United States. It has a very low five-year survival rate (< 5%) in the United States as well as in the world (about 9%). The current gemcitabine-based therapy soon becomes ineffective because treatment resistance and surgical resection also provides only selective benefit. Signature mutations in pancreatic cancer confer chemoresistance by deregulating the cell cycle and promoting anti-apoptotic mechanisms. The stroma-rich tumor microenvironment impairs drug delivery and promotes tumor-specific immune escape. All these factors render the current treatment incompetent and prompt an urgent need for new, improved therapy. In this review, we have discussed the genetics of pancreatic cancer and its role in tumor evolution and treatment resistance. We have also evaluated new treatment strategies for pancreatic cancer, like targeted therapy and immunotherapy.

## INTRODUCTION

Pancreatic cancer is a fatal disease that currently ranks third in the list of cancer-related deaths in the United States after lung cancer and colon cancer^[1]^. It has a low five-year survival rate of < 5% and a poor patient prognosis^[2]^. Different factors contribute towards the poor prognosis of pancreatic cancer such as lack of early stage-specific symptoms, dearth of definite screening tests, shortage of biomarkers, lack of effective therapy and acquired resistance^[3,4]^. The estimates show that by 2030 pancreatic cancer will be the second most common cause of cancer-related deaths in the United States, just after lung cancer^[4]^.

Pancreatic cancer can originate in either the exocrine or endocrine portion of the organ. The exocrine pancreatic cancer includes pancreatic ductal adenocarcinoma (PDAC), which is also the most commonly detected histological type in the clinic (~90% patients)^[5]^ and displays histology of the ductal cells of the pancreas, hence the name^[6]^. Other less common forms include acinar cell carcinoma, solid pseudopapillary tumors, serous cystadenoma, and pancreatoblastoma, *etc*.^[7,8]^. Pancreatic endocrine tumors originate in the endocrine glands of the pancreas. These tumors are rare and makeup < 5% of all pancreatic cancer cases^[9]^. Older age (> 50 years) is the major risk factor associated with pancreatic cancer^[4]^. Other risk factors include smoking (15%−30%), obesity (16%), diabetes mellitus, family history (5%−10%)^[4,10]^, and heavy alcohol consumption^[11]^. Some hereditary diseases like, Peutz-Jeghers syndrome, Lynch syndrome, and pancreatitis also raise the risk of pancreatic cancer^[12]^. The current treatment modalities provide a median survival of only 6 months^[3]^. Surgery combined with radiation and/or chemotherapy (preoperative or post-operative) is the only treatment option for patients diagnosed at advanced stages, which prolongs survival by 20%−25% in eligible patients^[13,14]^. Therefore for patients with metastatic disease, chemotherapy remains the only alternative^[15]^. This comprehensive review focuses on different gene mutations, therapeutic resistance, and the current treatment modalities for pancreatic cancer.

## ROLE OF SIGNATURE MUTATIONS IN PANCREATIC CANCER

Pancreatic cancer involves around 63 genetic mutations, bulk of which are point mutations. These mutations contribute to the dysregulation of at least 12 signaling pathways that are frequently altered in pancreatic tumors^[16]^. This causes heterogeneity in pancreatic tumors leading to aggressiveness and lack of targeted therapy. Pathological and molecular analysis of pancreatic tumors has identified the following signature mutations; mutations in *KRAS*, *TP53*, *CDKN2A*, and *SMAD4* genes^[4,7]^. These genetic lesions fuel uncontrolled growth and survival of pancreatic cancer cells by deregulating the cell cycle during different phases of pancreatic tumor development. These mutations also contribute to therapeutic resistance^[17]^. These signature mutations occur temporally over the course of pancreatic tumor development and steer the progress from PanIN to adenocarcinoma^[2]^. Table 1 shows the signature mutations involved in different stages of pancreatic cancer.

The driver mutation happens in *KRAS* of the normal pancreatic cells. As the disease progresses, mutations in *KRAS* accumulate and is thus observed in > 90% of pancreatic cancer cases^[7]^. *KRAS* is also frequently mutated in other human cancers (~85%) like colorectal (52%) and lung (31%) adenocarcinomas^[34]^.

Given the central role of KRAS in the initiation, growth, and progression of pancreatic cancer, it is imperative to target this oncogene. The use of KRAS as a therapeutic target for pancreatic cancer has been studied extensively in recent years. Several strategies have been developed to target mutant KRAS protein either genetically or using small molecules after *in silico* and *in vitro* screenings and assays^[35]^. In pancreatic cancer KRAS is known to upregulate Raf-MEK-ERK and PI3K/Akt signaling pathways^[18]^ which promote cell growth and survival of pancreatic cancer cells. The constitutive Ras signaling in pancreatic cancer cells causes aberrant activation of ERK, which facilitates the process of cell proliferation and tumor initiation^[36]^. In conclusion, constitutive activation of Raf-MEK-ERK signaling by mutant KRAS leads to increased levels of G1 cyclins that confer a survival advantage to these cells. This drives pancreatic carcinogenesis by inducing PanIN formation.

As pancreatic cancer approaches the low grade PanIN stage (PanIN 1A and 1B), mutations are acquired in *CDKN2A* gene in the form of homozygous deletions, mutations, or promoter hyper-methylation^[7,37]^. *CDKN2A* codes for p16 (INK4A) tumor suppressor that limits G1 to S transition by inhibiting the formation of Cyclin D1-CDK4/6 complex. Loss of p16 function is observed in 80%−90% of pancreatic cancer cases^[23]^ and also associates with poor patient prognosis^[38]^. P16 has been shown to counteract activated KRAS in normal fibroblasts by inducing premature senescence^[21,22]^. Therefore, it is speculated that pancreatic cancer cells lose p16 activity in order to gain the survival advantage offered by mutant *KRAS*. Additionally, loss of p16 has been implicated in chemoresistance^[39]^.

During the stage of medium grade PanIN (PanIN2), inactivating mutations in *TP53* are acquired. These are usually missense mutations that occur in the DNA binding domain of p53 and are encountered in about 50% of pancreatic cancer patients^[23]^. In pancreatic cancer, inactivation of p53 results in excessive genomic instability, which is often observed in this disease. Therefore, in the event of p53 inactivation, the pancreatic cancer cells accumulate any genetic abnormalities inflicted upon them. Loss of function of p53 also leads to chemoresistance to gemcitabine by preventing DNA damage-induced apoptosis (discussed in later sections). The majority of mutations found in PDAC for *TP53* gene are missense mutations leading to stable and highly expressed mutant p53 proteins^[40]^. A recent study showed that mutant p53 interacts with CREB1 upon KRAS activation, which hyperactivates several pro-metastatic transcriptional networks that drive PDAC metastasis^[41]^. Another study showed that upregulation of platelet-derived growth factor (PDGF) receptor beta mediates mutant p53 to drive the invasive phenotype of PDAC^[42]^.

At the stage of high-grade PanIN (PanIN3), *SMAD4* also gets altered. It is seen mutated or deleted in around 55% of pancreatic cancers and is also correlated with poor patient prognosis^[27]^. SMAD4 is a transcriptional regulator that is a key component in the transforming growth factor β (TGFβ) pathway. Since TGFβ signaling blocks cell growth and promotes differentiation, it is often mutated in cancers^[43,44]^. One of the functions of TGFβ is to cause G1 phase cell cycle arrest by inducing the expression of p27 (CKI) and prevent its degradation by downregulating Skp2 protein levels^[45]^. Therefore, it can be understood that the inactivation of Smad4 in pancreatic cancer cells removes p27 protein from the equation contributing to the disabling of G1/S checkpoint.

**Figure 1.**
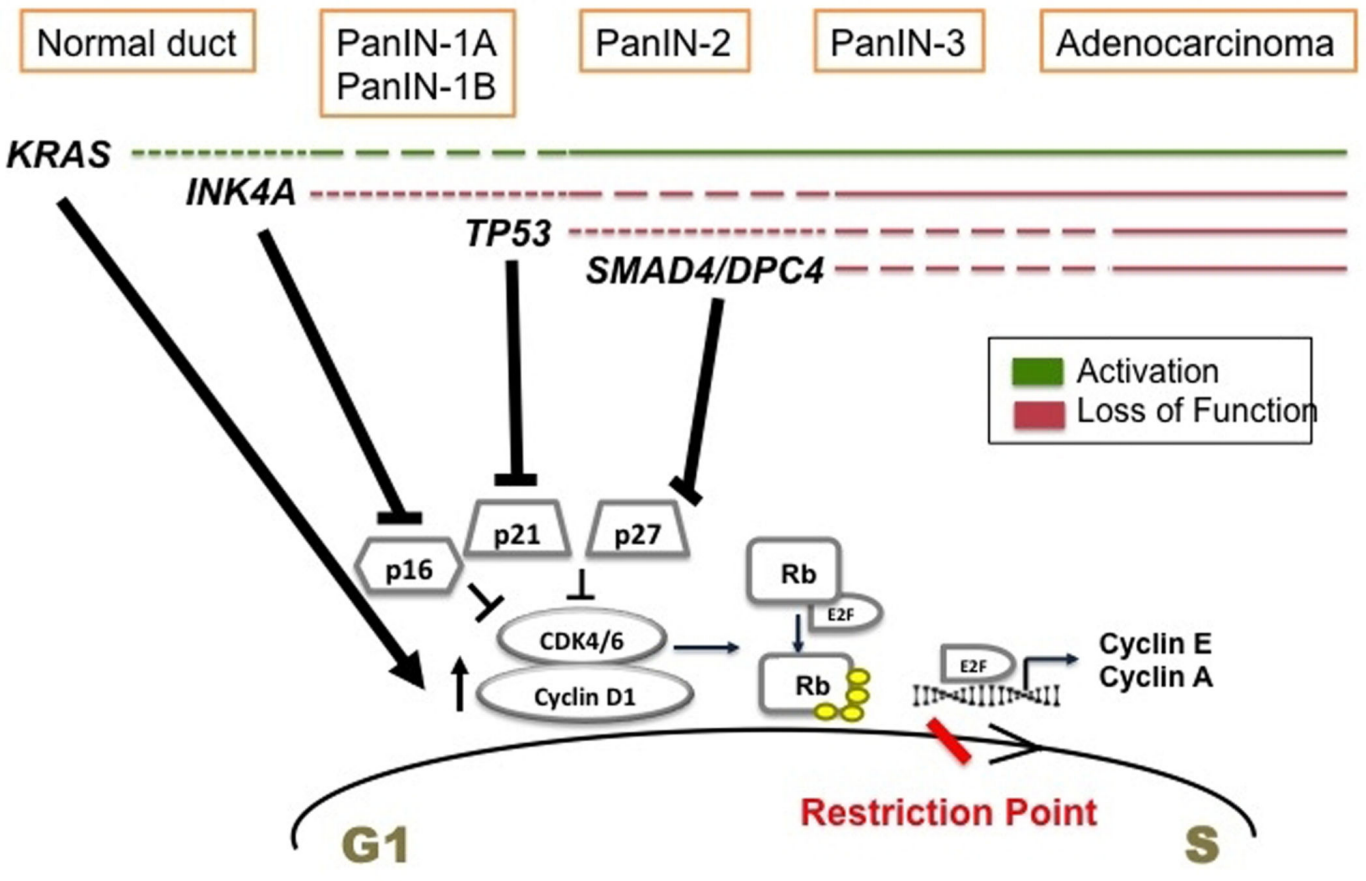
Temporal occurrence of signature mutations in pancreatic cancer and its effect on G1 to S transition. Driver mutations occur in *KRAS* of normal pancreatic cells initiating tumor formation. These mutations promote cell proliferation by upregulating cyclin D1. During the stages of low grade PanIN, *INK4A* mutations are acquired. It inactivates p16 tumor suppressor. As the tumor progresses to high grade PanIN, *TP53* and *SMAD4* are mutated mediating the inactivation of p21 and p27 CKIs. All these events deregulate G1 to S transition promoting uncontrolled proliferation and pancreatic cancer proceeds to full blown adenocarcinoma.

In conclusion, all the above-described mutations deregulate the cell cycle in pancreatic cancer cells, mainly at G1 to S transition [Figure 1]. *KRAS* mutation upregulates cyclin D1 (G1 cyclin), whereas the mutations in *CDKN2A*, *TP53*, and *SMAD4* inactivate the tumor suppressors, p16, p21, and p27 respectively. All these events render the G1/S checkpoint dysfunctional and set the stage for malignant transformation.

## PANCREATIC CANCER AND THERAPEUTIC RESISTANCE

Adjuvant chemotherapy after surgical resection remains the primary treatment for early pancreatic cancer patients. For the past two decades, gemcitabine (gemzar^®^) has been the mainstay of pancreatic cancer treatment. Gemcitabine was approved by FDA in 1996 on the basis that it increased survival in five-fold more patients over 5-FU (5-Fluorouracil), the previously used drug for pancreatic cancer chemotherapy^[46]^. Here we will discuss the metabolic actions of gemcitabine and the mechanisms involved in the therapeutic resistance of gemcitabine.

### Metabolism of gemcitabine

**Figure 2.**
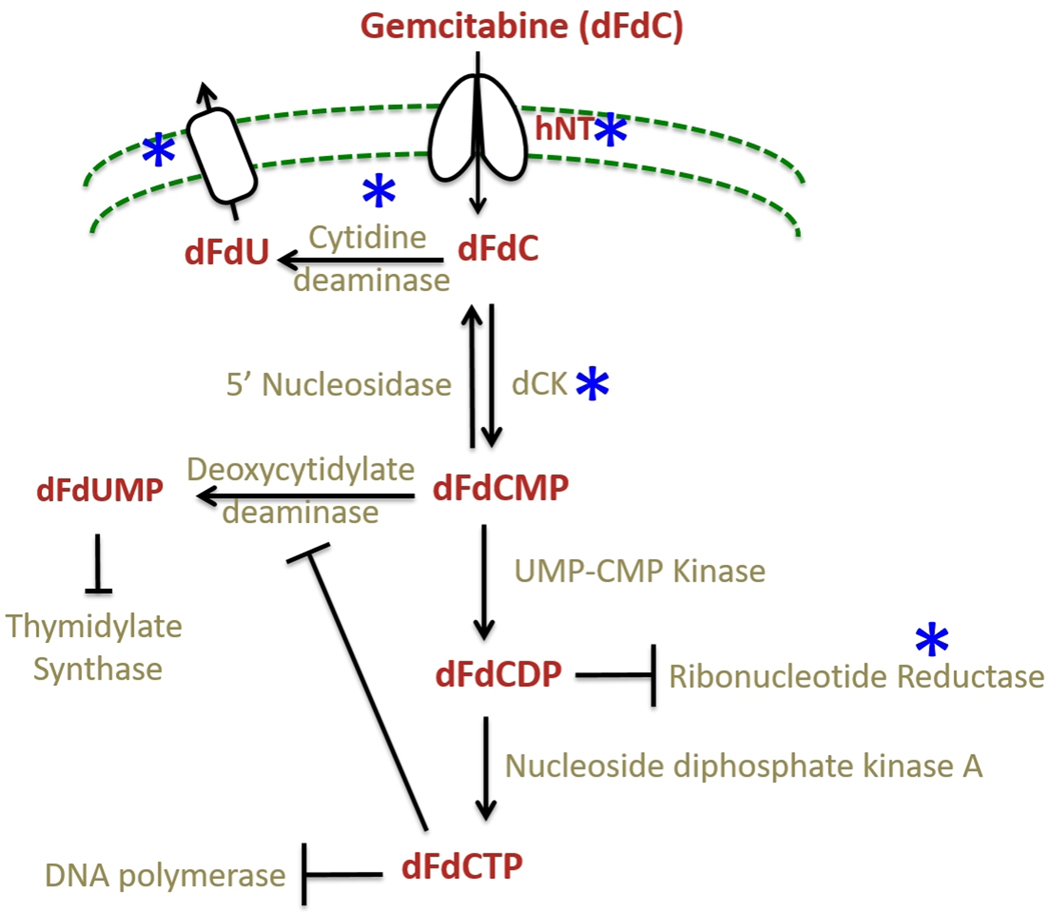
The gemcitabine metabolism and its mechanism of resistance. Gemcitabine is taken into cells by nucleoside transporters and converted by a series of reactions into dFCTP. It is incorporated into replicating DNA resulting in chain termination. The incorporated dFdCTP leads to dislodgement of DNA polymerase one nucleotide downstream of the dFdCTP. This extra nucleotide masks the break site and makes it imperceptible to the DNA repair enzymes leading to DNA damage^[49]^; whereas dFdCDP inhibits ribonucleotide reductase (RNR) enzyme leading to reduced pools of dCTP thus creating a positive feedback loop ensuring gemcitabine incorporation. The steps affected by the resistance mechanisms are starred (*) in blue.

Gemcitabine is a deoxycytidine analog that functions by interfering with the DNA synthesis pathway and eventually inducing apoptosis. Gemcitabine is a prodrug that is taken up into the cells mainly by two human nucleoside transporters, equilibrative nucleoside transporters (ENT), and concentrative nucleoside transporters (CNT)^[47]^. Inside the cells, it gets converted into dFdCDP and dFdCTP by a series of reactions initiated by deoxycytidine kinase (dCK) enzyme, which obstructs DNA replication by inhibiting DNA polymerase^[46,48]^, culminating in DNA damage-induced apoptosis^[46]^. Metabolism of gemcitabine and the components affected by resistance mechanisms are shown in Figure 2 and summarized in Table 2.

### Mechanisms of gemcitabine resistance

It has been observed that pancreatic cancer patients acquire resistance to gemcitabine therapy soon after the starting of treatment resulting in poor patient response^[49]^ (highlighted in Figure 2). The mechanisms of gemcitabine resistance can be classified into two categories: (1) mechanisms that impede gemcitabine metabolism and (2) mechanisms that intercept gemcitabine-induced apoptosis^[49]^. The first category involves mechanisms like downregulation of CNT1 and ENT1 transporters in pancreatic cancer cells to decrease the uptake of gemcitabine^[50,51]^. Overexpression of cytidine deaminase (CDA) is also observed in pancreatic cancer cells along with MRP-1 (multidrug resistance-associated protein) transporter responsible for causing an efflux of clinically relevant drugs^[52]^. Another mechanism is the downregulation of dCK enzyme, which prevents the breakdown of gemcitabine into its active metabolites. Studies have shown that levels of dCK correlate with the overall survival of pancreatic cancer patients^[53]^. Increased RNR expression is associated with sustained dCTP pools and inhibition of gemcitabine-incorporation^[54,55]^. The second category of gemcitabine resistance mechanisms involves upregulation of survival pathways like PI3K/Akt and unfolded protein response (UPR) interfering with gemcitabine induced apoptosis^[56,57]^. PI3K upregulation is associated with poor patient prognosis^[58,59]^ and is known to prevent gemcitabine induced apoptosis^[60]^. The inhibition of PI3K/Akt pathway has shown promise in sensitizing pancreatic cancer cells towards apoptosis induced by gemcitabine as well as other chemotherapeutics both *in vitro* and *in vivo*^[61]^. Other mechanism includes inactivation of p53 tumor suppressor protein by mutations resulting in inhibition of DNA damage-induced apoptosis (discussed previously).

### Role of desmoplasia in pancreatic cancer chemoresistance

Desmoplasia or inflammatory fibrotic reaction is considered as the histological hallmark of pancreatic cancer, which makes up to 90 percent of total tumor volume^[62]^. The pancreatic stroma is composed of both cellular and acellular components; the cellular components are fibroblasts, myofibroblasts, pancreatic stellate cells (PSCs), and immune cells, and acellular components are blood vessels, extracellular matrix (ECM), cytokines, and growth factors^[63]^. The desmoplastic stroma is primarily composed of cancer-associated fibroblasts (CAFs), immune cells, small blood vessels, and ECM^[64]^. In normal pancreatic tissue, the PSCs are found in a quiescent state^[64]^. Upon tissue injury, the PSCs are activated by pancreatic tumor cells and acquire a myofibroblast-like appearance^[63]^. Factors like aberrant TGFβ signaling due to *SMAD4* deletion combined with *KRAS* mutation, PDGF, tumor necrosis factor α, and several interleukins (IL-1, 6 and 10) can initiate the desmoplastic reaction. The pancreatic tumor cells secrete these factors, which bind to their respective receptors present on the PSC and activate them by their specific signaling resulting in increased ECM deposition. The activated PSCs create an autocrine loop and promote tumor growth and migration^[62,64]^. CAFs also overexpress SMO and have a hyperactive Hh pathway that further contributes to their maintenance^[65]^.

The activated PSCs or CAFs also secrete ECM components like collagens, laminins, fibronectin, hyaluronic acid (HA), *etc*. This results in the development of dense stroma around the tumor that acts as a structural barrier to drug delivery^[66,67]^. In addition, stromal fibroblasts lead to increased interstitial fluid pressure (IFP) by acquiring contractile properties and increasing contraction of the interstitial matrix, thus posing a physical barrier to drug delivery in pancreatic tumors^[67,68]^. It can be understood that desmoplasia is another contributory factor to drug resistance in pancreatic cancer. Several stromal components like CAFs, HA, collagen (type 1), *etc*., also exclusively contribute to gemcitabine resistance by various mechanisms promoting apoptosis resistance^[69]^. The pancreatic stroma also induces tumor microenvironment-associated stresses which upregulate UPR and promote survival in pancreatic cancer cells. Therefore, the pancreatic stroma is another attractive target for therapy.

Till date, various studies have targeted different components of the pancreatic stroma. The anti-fibrotic drug pirfenidone, approved for the treatment of pulmonary fibrosis, inhibits fibroblasts and production of TGFβ, PDGF, and collagen type 1 in PDAC mouse model^[70]^. Another study in *KPC* mouse model demonstrated that targeting HA lowers IFP in the tumors and inhibits tumor growth due to improved drug delivery^[71,72]^. A phase 1b clinical trial was done to test the safety and efficacy of Pegylated recombinant human hyaluronidase (PEGPH20) and gemcitabine combination in stage IV PDAC patients. PEGPH20 depleted interstitial HA, which caused a decrease in IFP and improvement in drug delivery^[73]^. In 2005, the FDA approved the use of nanoparticle- albumin-bound paclitaxel (nab^®^-paclitaxel) (trade name: ABRAXANE) for the treatment of the pancreatic cancer. It was later shown that nab-paclitaxel causes disruption of pancreatic stroma by softening the tumor by decreasing its CAF content^[74]^. The MPAC trial in 2013 by Von Hoff *et al*.^[75]^ combined nab-paclitaxel with gemcitabine for the treatment of metastatic pancreatic cancer patients showing improved overall survival over gemcitabine alone. In the same year, nab^®^-paclitaxel plus gemcitabine combination received FDA approval for the treatment of metastatic pancreatic cancer. Various studies targeting different components of pancreatic stroma are summarized in Table 3.

## BARRIERS TO CURRENT CHEMOTHERAPY IN PANCREATIC CANCER

Pancreatic cancer is a deadly disease with disappointing statistics. The current treatment options are limited and largely ineffective. The reasons for therapy failure in pancreatic cancer are multifold and need to be considered while designing new therapies. This section highlights some of the inherent features of pancreatic tumors that present a barrier to chemotherapy in general. The signature mutations encountered in pancreatic cancer are not only responsible for its progression but also chemoresistance. As highlighted in previous sections, driver mutations in *KRAS* result in aberrant activation of downstream signaling pathways like Raf-MEK-ERK MAPK and PI3K/Akt signaling^[18]^. These survival pathways promote resistance to chemotherapy by causing apoptotic resistance through the upregulation of anti-apoptotic proteins in the cell in response to chemotherapeutic agents. For example, the ERK mediates induction of anti-apoptotic proteins like Bcl-2, Mcl-1, and Bcl-X(L), which prevent chemotherapy-induced apoptosis in pancreatic cancer cells^[83,84]^. Inactivating mutations in *TP53* tumor suppressor lowers the ability of the cell to sense the DNA damage induced by gemcitabine incorporation, therefore, particularly inhibiting gemcitabine-induced apoptosis in pancreatic cancer cells^[23]^. Additionally, p21 (CKI) is not induced by a non-functional p53, and cell cycle is not arrested for the DNA repair^[85]^. Similarly, mutations in *INK4A* and *SMAD4* cause the inactivation of p16 and p27 (CKIs) tumor suppressors respectively, and lead to an uninterrupted cell cycle^[39,44]^. Due to the highly proliferative and secretory nature of pancreatic cancer cells, UPR pathway is also expected to play a protective role in these cells by maintaining protein folding quality control^[86]^. The desmoplastic microenvironment of pancreatic tumors inflicts stresses like hypoxia and nutrient starvation which upregulate UPR in these cells to prevent stress-induced apoptosis^[87]^. Furthermore, the presence of dense stroma around the tumor poses a physical barrier to drug delivery^[66]^. The individual stromal components also induce gemcitabine resistance by inhibiting the apoptosis of pancreatic cancer cells^[69]^. Several intrinsic mechanisms also exist in pancreatic cancer cells, which interfere with the metabolism of gemcitabine and prevent its incorporation into the DNA. These mechanisms include decreased expression of NTs to prevent gemcitabine uptake into the cells. For example, inactivation of dCK enzyme to prevent gemcitabine breakdown, upregulation CDA to promote metabolic deactivation of gemcitabine and its consequent efflux through overexpressed ABC pumps, and upregulation of RNR to counteract the effect of gemcitabine by maintaining the dCTP pools in the cell^[46,50–55]^. In all, it is evident that pancreatic tumors have evolved diverse mechanisms to protect themselves from gemcitabine treatment and chemotherapy in general. Therefore, to efficiently treat pancreatic cancer, new improved therapies need to be designed that can cross the presented hurdles.

## DIFFERENT STRATEGIES TO COMBAT PANCREATIC CANCER

### Immunotherapy

Immunotherapy is the latest addition to the treatment design for solid tumors. It involves targeting immune checkpoint molecules CTLA-4 (Cytotoxic T-lymphocyte associated antigen 4), PD-1 (Programmed cell death-1), PD-L1 (Programmed cell death ligand-1) using monoclonal antibodies. CTLA-4 inhibitors have been tested in melanoma, renal cell cancer, NSCLC, SCLC, ovarian cancer, *etc*.^[88]^. PD-L1 expression has been detected by IHC in a variety of solid tumors, including pancreatic cancer^[89]^. The desmoplastic microenvironment of pancreatic tumors protects them from host innate immunity in many ways. Pancreatic tumor stroma has been shown to have an activated CD40 pathway which is involved in establishing tumor-specific T cell immunity^[90]^. Reports have also shown that the prevalence of CD4+ Th2 cells in the pancreatic tumor stroma is associated with poor patient prognosis^[91]^. However, the presence of CD4+ and CD8+ TIL together is an indicator of a good prognosis in surgically resected PDAC patients^[92]^.

Several immune checkpoint inhibitors have been tested in pancreatic cancer, alone and combined with radiation and chemotherapy. Ipilimumab, a CTLA-4 inhibitor, alone and in combination with gemcitabine or Nivolumab (PD-1 inhibitor) and has been tested in unresectable/locally advanced/ metastatic stage III or IV pancreatic cancer^[93]^. Ipilimumab alone did not show any improvement in patient survival^[94]^. However, Ipilimumab and gemcitabine combination had an OS of 8.5 months^[95]^. Other CTLA-4 inhibitors like Tremelimumab and PD-1 inhibitors like Pembrolizumab and Atezolizumab have also been tested alone and in combination in advanced pancreatic cancer. These studies have shown varying OS in different phases of clinical trials^[93]^.

Anti-cancer vaccines are the other therapeutic modalities that have been tried in pancreatic cancer. Several kinds of vaccines exist like whole-cell vaccines, peptide-based vaccines, dendritic cell vaccines, DNA vaccines (plasmid vaccines, virus-based vaccines, bacterial vectors, and yeast-based recombination vaccines), and mRNA vaccines. Various vaccines are being tested in clinical trials at Pre-clinical/Phase I/Phase II stages for metastatic pancreatic cancer. Some of these vaccines include OCV-C01, GVAX, synthetic Ras peptides, Mucin-1 peptides, *etc*.^[96]^. OCV-C01, in combination with gemcitabine, has shown better DFS of 15.8 months over gemcitabine alone (12 months)^[97]^. GVAX is a whole tumor vaccine that is engineered to express GM-CSF (granulocyte macrophage- colony-stimulating factor). This causes induction of APC antigen uptake and T cell priming. In at least 5 clinical trials, GVAX (in combination) showed the anti-tumor response in tumors and increased OS in patients with low or minimum toxicity^[98]^. A combination of CTLA-4 inhibitor, Ipilimumab, and cancer vaccine, GVAX has also been tested in previously treated advanced pancreatic cancer. The patients in which OS > 4.3 months showed an increase in peak mesothelin-specific T-cells and T-cell repertoire^[99]^.

The above-discussed studies suggest that although immunotherapy is still in its preliminary stages, it holds a strong potential to be developed as a therapeutic for pancreatic cancer treatment.

### Poly (ADP-ribose) polymerase inhibitors

Pancreatic cancer is the third most common cancer related to early-onset mutation in the breast cancer (*BRCA*) gene. Approximately 4%−7% of patients with PDAC have germline BRCA1/2 mutations (gBRCA1/2)^[100,101]^. These mutations have potential therapeutic implications as they confer increased sensitivity to platinum-based chemotherapy and poly (ADP-ribose) polymerase inhibitors (PARPi)^[102]^. Cancer cells with mutations that prevent homologous recombination repair, such as BRCA1/2 loss-of-function mutations, are often synthetically lethal with PARPi due to significantly lower DNA damage response^[103]^. PARPi causes unrepaired accumulation of single-strand DNA breaks, which eventually culminate into double-strand breaks, causing the death of the BRCA1/2-mutant cancer cells^[104]^. PARPi have become the most commonly used drug to target BRCA mutations. The use of PARPi in PDAC is an active area of investigation which is developing from being used as monotherapies to combination therapy with other classes of therapeutic agents. Olaparib, a small molecule PARPi, has proven efficacy against germline BRCA-mutated metastatic pancreatic cancer patients^[105]^; and is the only accepted PARPi for clinical application in pancreatic cancer^[106]^. Several trials are in the clinic using olaparib as monotherapy in advanced disease of PDAC^[104]^. In addition to PARPi alone, clinical trials are currently underway to evaluate PARPi combinations with other classes of therapies causing DNA damage in pancreatic cancer patients^[107]^.

Gemcitabine is widely used as a radiosensitizer for PDAC treatment and other cancers^[108,109]^. It is known to induce tumor cells S-phase arrest and thus sensitize cells to DNA damage^[108]^. PARPi could sensitize cells to exogenous DNA damage inducer treatment, such as irradiation in pancreatic cancer cell’s^[110]^ or gemcitabine in non-small-cell lung cancer^[111]^. Combination treatment of PARPi- olaparib with gemcitabine and proton therapy significantly enhanced tumor response and progression-free survival in pancreatic cancer mice model^[112]^. Taken together, these studies provide crucial evidence that a combination of PARPi with gemcitabine for radiosensitization could be used as an improved therapeutic regimen for overcoming the therapeutic resistance in pancreatic cancer.

### Cancer-associated fibroblasts

CAFs have emerged as key players in mediating drug resistance due to their presence within the PDAC tumor, along with their secreted factors. CAFs and their generated ECM can function as a physical barrier and thus prevent efficient drug delivery^[113]^. Targeting CAFs is becoming a promising therapeutic strategy owing to their involvement in the progression of tumorigenesis and drug resistance^[113]^, their genetic stability and relative abundance among stromal cells^[114]^. Currently, numerous clinical trials based on CAF-directed anticancer therapies with a goal of either normalizing CAFs or reduce their secretion are going on^[115]^.

CAFs have been shown to exert immunosuppressive effects through different mechanisms^[116–118]^. Francescone *et al*.^[119]^, investigated the role of CAFs in PDAC tumorigenesis. They showed that Netrin G1 expression in CAFs creates an immunosuppressive microenvironment that inactivates natural killer (NK) cells and protects PDAC cells from NK cell-mediated death^[119]^. Their data suggest an important role of CAFs in the microenvironment (i.e., ECM) in PDAC cell survival. Fibroblast Activation Protein (FAP) is frequently (90%) expressed, predominantly in CAFs, with pancreatic cancer patients^[120]^. High expression of FAP is associated with shorter overall survival and disease-free survival in pancreatic cancer patients. Several clinical trials targeting FAP in metastatic pancreatic cancer and other cancers are underway^[121]^. A recent study highlighted the importance of stromal macropinocytosis to support CAF cell fitness and providing amino acids in sustaining PDAC cell survival^[122]^. Macropinocytosis is a form of endocytosis that mediates non-selective fluid-phase uptake and represents a survival strategy in PDAC patients. Targeting macropinocytosis is another potential area to explore in pancreatic cancer since the pancreatic tumors exhibit high levels of macropinocytosis^[123]^, and selective disruption of macropinocytosis in CAFs helps suppress PDAC tumor growth^[122]^. All these studies reinforce the importance of considering the stroma as a promising therapeutic option in PDAC.

## CONCLUSION

Pancreatic cancer is a complex disease that has developed many shields to combat therapy. Overcoming gemcitabine resistance has been the focus of many conventional therapies, including adjuvant therapy, neoadjuvant therapy, targeted therapy as well as immunotherapy^[124]^. Within the last decade, several clinical trials have shown benefit in pancreatic cancer patients after using gemcitabine with other agents. For example, the patients treated with gemcitabine/nab-paclitaxel had an overall survival of 5.5 months compared to 3.7 months for the gemcitabine alone group^[75]^, and patients treated with 5-fluorouracil/leucovorin with irinotecan and oxaliplatin (FOLFIRINOX) survived for 6.4 months compared to 3.3 months survival of gemcitabine alone group^[125]^. These studies have shown improved survival outcomes in patients, but the improvement is still not huge. Despite the improved prognosis of advanced pancreatic cancer using the above treatments, the development of chemoresistance severely limits the effectiveness of the chemotherapy^[56]^. Other factors like unavailability of efficient screening method, lack of specific symptoms or biomarkers, and aggressive nature of this disease also contribute to the difficulty treating pancreatic cancer. Most of the cases are diagnosed only after metastasis, which not just limits surgical resection, but also lowers the chances of survival. Therefore, in order to efficiently treat this disease, a bi-directional strategy needs to be followed. One direction should aim at early detection of the tumor, and the other direction should focus on designing efficient therapy with all the resistance mechanisms in mind. Thus, it is important to investigate new methods and targets that can act as a catalyst in pancreatic cancer treatment.

## Figures and Tables

**Table 1.** Most commonly occurring mutations in different stages of pancreatic cancer

Mutations	Type	%	Stage	Effects	Ref.

*KRAS*	Activating	> 90%	PanIN	Promote cell growth and survival	[7,18–20]
*P16/CDKN2A*	Homozygous deletions, mutations or promoter hyper-methylation (Inactivated)	> 90%	PanIN 1A and 1B	Counter-activated K-Ras in normal fibroblasts by inducing premature senescence	[7,20–22]
*TP53*	Inactivating	50%	PanIN2	Cell cycle arrest and cell apoptosis	[20,23]
*SHH*	Activating	70%	PanIN1 and PanIN2	Shh is a downstream effector of oncogenic KRAS^G12D^ in pancreatic cancer development	[24–26]
*SMAD4*	Inactivating	55%	PanIN3	Encodes the transforming growth factor beta (TGF β) signaling pathway	[20,27]
*BRCA1/2*	Deleterious germline mutations	5%–9%	PanIN3	Homologous repair deficiency	[26,28,29]
*AKT*	Amplification	10%–20%	Stage I and Stage IV	Enhances the tumorigenicity and *in vivo* invasive potential of human pancreatic carcinoma cell line	[20,30,31]
*PIK3CA*	Amplification	3%–5%		Required for KRAS^G12D^-induced pancreatic tumorigenesis	[32,33]

**Table 2.** Mechanisms of gemcitabine resistance

Factors affecting gemcitabine metabolism		
Protein	Alteration	Effect
CNT1/ENT1 transporters	Downregulation	Decreased gemcitabine uptake
CDA/ MRP-1	Overexpression	Increased efflux of gemcitabine
dCK	Downregulation	Decreased gemcitabine breakdown into its metabolites
RNR	Overexpression	Maintain dCTP pools, decreased gemcitabine incorporation into DNA
Factors inhibiting gemcitabine induced apoptosis		
Signaling modality	Alteration	Effect
PI3K/Akt signaling	Upregulation	Increased cell survival
UPR	Upregulation	Increased cell survival
*P53* mutation	Inactivation	Decreased DNA damage induced apoptosis

**Table 3.** Studies showing targeting different components of pancreatic stroma

Drug/targeted agent	Effects/outcomes	Study model	Ref.

Pirfernidone (anti-fibrotic agent)	Inhibited fibroblasts and production of TGFβ, PDGF and collagen type 1	Mouse PDAC	[70]
Hyaluronic acid (HA)	Lowered interstitial fluid pressure in the tumors and inhibited tumor growth due to improved drug delivery	*KPC* mice	[71,72]
PEGylated hyaluronidase a (PEGPH20, an enzyme that temporarily degrades HA) plus gemcitabine	Depleted interstitial HA which caused a decrease in IFP and improvement in drug delivery	Stage IV PDAC patients	[73]
Nab^®^-paclitaxel plus gemcitabine	Disrupted pancreatic stroma by softening the tumor by decreasing its CAF content	Advanced pancreatic cancer patients	[74]
TNP 470 (a synthetic analogue of the fungus derived bioactive agent fumagillin)	Significantly decreased tumor size and spread in orthotopic xenograft models	*PDAC* mice	[76]
I PI-926 (a Shh inhibitor)	Enhanced delivery of gemcitabine through depletion of stromal tissue and increase in vascular density	*PDAC* mice	[77]
Deshydroproline (competitive inhibitor of proline dehydrogenase 1)	Decrease in PDAC cell proliferation, survival and tumor volumes	*In vitro,* mice PDAC	[78,79]
Fasudil (inhibitor for Rho-associated protein kinases (ROCKs) 1 and 2 genes)	Improved animal survival along with increased levels of a surrogate marker for tissue collagen	*KPC* mice	[79,80]
VS-4718 (Focal Adhesion Kinase small- molecule inhibitor)	Reduced tumor fibrosis, progression, metastasis, infiltrating immunosuppressive myeloid populations and improved animal survival	*KPC* mice	[79,81]
7rh (small-molecule inhibitor of Discoidin domain receptor 1)	Attenuated PYK2 and PEAK1 pro-tumor signaling and decreased migratory capacity of cancer cells *in vitro,* along with improved animal survival	*In vitro,* orthotopic and KPC mice	[79,82]

## References

[1] SiegelRL, MillerKD, JemalA. Cancer statistics, 2020. CA Cancer J Clin 2020;70:7–30. DOI PubMed31912902 10.3322/caac.21590

[2] FokasE, O’NeillE, Gordon-WeeksA, MukherjeeS, McKennaWG, MuschelRJ. Pancreatic ductal adenocarcinoma: from genetics to biology to radiobiology to oncoimmunology and all the way back to the clinic. Biochim Biophys Acta 2015;1855:61–82. DOI PubMed25489989 10.1016/j.bbcan.2014.12.001

[3] Garrido-LagunaI, HidalgoM. Pancreatic cancer: from state-of-the-art treatments to promising novel therapies. Nat Rev Clin Oncol 2015;12:319–34. DOI PubMed25824606 10.1038/nrclinonc.2015.53

[4] KleeffJ, KorcM, ApteM, Pancreatic cancer. Nat Rev Dis Primers 2016;2:16022. DOI PubMed27158978 10.1038/nrdp.2016.22

[5] AdamskaA, DomenichiniA, FalascaM. Pancreatic ductal adenocarcinoma: current and evolving therapies. Int J Mol Sci 2017;18:1338. DOI PubMed PMC28640192 10.3390/ijms18071338PMC5535831

[6] LüttgesJ, HahnS, KlöppelG. Where and when does pancreatic carcinoma start? Med Klin (Munich) 2004;99:191–5. DOI PubMed15085289 10.1007/s00063-004-1028-3

[7] BardeesyN, DePinhoRA. Pancreatic cancer biology and genetics. Nat Rev Cancer 2002;2:897–909. DOI PubMed12459728 10.1038/nrc949

[8] BellizziAM, FrankelWL. Pancreatic pathology: a practical review. Lab Med 2009;40:417–26.1. DOI

[9] RoC, ChaiW, YuVE, YuR. Pancreatic neuroendocrine tumors: biology, diagnosis,and treatment. Chin J Cancer 2013;32:312–24. DOI PubMed PMC23237225 10.5732/cjc.012.10295PMC3845620

[10] SalemAA, MackenzieGG. Pancreatic cancer: a critical review of dietary risk. Nutr Res 2018;52:1–13. DOI PubMed29764623 10.1016/j.nutres.2017.12.001

[11] GuptaS, WangF, HollyEA, BracciPM. Risk of pancreatic cancer by alcohol dose, duration, and pattern of consumption, including binge drinking: a population-based study. Cancer Causes Control 2010;21:1047–59. DOI PubMed PMC20349126 10.1007/s10552-010-9533-6PMC2883092

[12] BeckerAE, HernandezYG, FruchtH, LucasAL. Pancreatic ductal adenocarcinoma: risk factors, screening, and early detection. World J Gastroenterol 2014;20:11182–98. DOI PubMed PMC25170203 10.3748/wjg.v20.i32.11182PMC4145757

[13] WörmannSM, AlgülH. Risk factors and therapeutic targets in pancreatic cancer. Front Oncol 2013;3:282. DOI PubMed PMC24303367 10.3389/fonc.2013.00282PMC3831165

[14] BlissLA, WitkowskiER, YangCJ, TsengJF. Outcomes in operative management of pancreatic cancer. J Surg Oncol 2014;110:592–8. DOI PubMed25111970 10.1002/jso.23744

[15] HeinemannV Gemcitabine: progress in the treatment of pancreatic cancer. Oncology 2001;60:8–18. DOI PubMed11150902 10.1159/000055290

[16] JonesS, ZhangX, ParsonsDW, Core signaling pathways in human pancreatic cancers revealed by global genomic analyses. Science 2008;321:1801–6. DOI PubMed PMC18772397 10.1126/science.1164368PMC2848990

[17] MimeaultM, BrandRE, SassonAA, BatraSK. Recent advances on the molecular mechanisms involved in pancreatic cancer progression and therapies. Pancreas 2005;31:301–16. DOI PubMed16258363 10.1097/01.mpa.0000175893.04660.1b

[18] ShimizuT, TolcherAW, PapadopoulosKP, The clinical effect of the dual-targeting strategy involving PI3K/AKT/mTOR and RAS/MEK/ERK pathways in patients with advanced cancer. Clin Cancer Res 2012;18:2316–25. DOI PubMed22261800 10.1158/1078-0432.CCR-11-2381

[19] MoskalukCA, HrubanRH, KernSE. p16 and K-ras gene mutations in the intraductal precursors of human pancreatic adenocarcinoma. Cancer Res 1997;57:2140–3. PubMed9187111

[20] KaranikasM, EsempidisA, ChasanZT, Pancreatic cancer from molecular pathways to treatment opinion. J Cancer 2016;7:1328–39. DOI PubMed PMC27390608 10.7150/jca.15419PMC4934041

[21] SerranoM, LinAW, McCurrachME, BeachD, LoweSW. Oncogenic ras provokes premature cell senescence associated with accumulation of p53 and p16INK4a. Cell 1997;88:593–602. DOI PubMed9054499 10.1016/s0092-8674(00)81902-9

[22] BrookesS, RoweJ, RuasM, INK4a-deficient human diploid fibroblasts are resistant to RAS-induced senescence. EMBO J 2002;21:2936–45. DOI PubMed PMC12065407 10.1093/emboj/cdf289PMC126048

[23] RozenblumE, SchutteM, GogginsM, Tumor-suppressive pathways in pancreatic carcinoma. Cancer Res 1997;57:1731–4. PubMed9135016

[24] NakashimaH, NakamuraM, YamaguchiH, Nuclear factor-kappaB contributes to hedgehog signaling pathway activation through sonic hedgehog induction in pancreatic cancer. Cancer Res 2006;66:7041–9. DOI PubMed16849549 10.1158/0008-5472.CAN-05-4588

[25] LingJ, KangY, ZhaoR, KrasG12D-induced IKK2/β/NF-κB activation by IL-1α and p62 feedforward loops is required for development of pancreatic ductal adenocarcinoma. Cancer Cell 2012;21:105–20. DOI PubMed PMC22264792 10.1016/j.ccr.2011.12.006PMC3360958

[26] GuD, SchlotmanKE, XieJ. Deciphering the role of hedgehog signaling in pancreatic cancer. J Biomed Res 2016;30:353–60. DOI PubMed PMC27346466 10.7555/JBR.30.20150107PMC5044707

[27] TascilarM, SkinnerHG, RostyC, The SMAD4 protein and prognosis of pancreatic ductal adenocarcinoma. Clin Cancer Res 2021;7:4115–21. PubMed11751510

[28] LoweryMA, WongW, JordanEJ, Prospective evaluation of germline alterations in patients with exocrine pancreatic neoplasms. J Natl Cancer Inst 2018;110:1067–74. DOI PubMed PMC29506128 10.1093/jnci/djy024PMC6186514

[29] WongW, RaufiAG, SafyanRA, BatesSE, ManjiGA. BRCA mutations in pancreas cancer: spectrum, current management, challenges and future prospects. Cancer Manag Res 2020;12:2731–42. DOI PubMed PMC32368150 10.2147/CMAR.S211151PMC7185320

[30] ChengJQ, RuggeriB, KleinWM, Amplification of AKT2 in human pancreatic cells and inhibition of AKT2 expression and tumorigenicity by antisense RNA. Proc Natl Acad Sci U S A 1996;93:3636–41. DOI PubMed PMC8622988 10.1073/pnas.93.8.3636PMC39663

[31] RuggeriBA, HuangL, WoodM, ChengJQ, TestaJR. Amplification and overexpression of the AKT2 oncogene in a subset of human pancreatic ductal adenocarcinomas. Mol Carcinog 1998;21:81–6. PubMed9496907

[32] PayneSN, MaherME, TranNH, PIK3CA mutations can initiate pancreatic tumorigenesis and are targetable with PI3K inhibitors. Oncogenesis 2015;4:e169. DOI PubMed PMC26436951 10.1038/oncsis.2015.28PMC4632089

[33] SivaramN, McLaughlinPA, HanHV, Tumor-intrinsic PIK3CA represses tumor immunogenecity in a model of pancreatic cancer. J Clin Invest 2019;129:3264–76. DOI PubMed PMC31112530 10.1172/JCI123540PMC6668699

[34] PapkeB, DerCJ. Drugging RAS: know the enemy. Science 2017;355:1158–63. DOI PubMed28302824 10.1126/science.aam7622

[35] BuscailL, BournetB, CordelierP. Role of oncogenic KRAS in the diagnosis, prognosis and treatment of pancreatic cancer. Nat Rev Gastroenterol Hepatol 2020;17:153–68. DOI PubMed32005945 10.1038/s41575-019-0245-4

[36] ChambardJC, LeflochR, PouysségurJ, LenormandP. ERK implication in cell cycle regulation. Biochim Biophys Acta 2007;1773:1299–310. DOI PubMed17188374 10.1016/j.bbamcr.2006.11.010

[37] HuangL, GoodrowTL, ZhangSY, Klein-SzantoAJ, ChangH, RuggeriBA. Deletion and mutation analyses of the P16/MTS-1 tumor suppressor gene in human ductal pancreatic cancer reveals a higher frequency of abnormalities in tumor-derived cell lines than in primary ductal adenocarcinomas. Cancer Res 1996;56:1137–41. PubMed8640773

[38] GerdesB, RamaswamyA, ZieglerA, p16INK4a is a prognostic marker in resected ductal pancreatic cancer: an analysis of p16INK4a, p53, MDM2, an Rb. Ann Surg 2002;235:51–9. DOI PubMed PMC11753042 10.1097/00000658-200201000-00007PMC1422395

[39] SchmittCA, FridmanJS, YangM, A senescence program controlled by p53 and p16INK4a contributes to the outcome of cancer therapy. Cell 2002;109:335–46. DOI PubMed12015983 10.1016/s0092-8674(02)00734-1

[40] MortonJP, TimpsonP, KarimSA, Mutant p53 drives metastasis and overcomes growth arrest/senescence in pancreatic cancer. Proc Natl Acad Sci U S A 2010;107:246–51. DOI PubMed PMC20018721 10.1073/pnas.0908428107PMC2806749

[41] KimMP, LiX, DengJ, Oncogenic. KRAS :recruits an expansive transcriptional network through mutant p53 to drive pancreatic cancer metastasis. Cancer Discov 2021. DOI PubMed10.1158/2159-8290.CD-20-1228PMC833888433839689

[42] WeissmuellerS, ManchadoE, SaborowskiM, Mutant p53 drives pancreatic cancer metastasis through cell-autonomous PDGF receptor β signaling. Cell 2014;157:382–94. DOI PubMed PMC24725405 10.1016/j.cell.2014.01.066PMC4001090

[43] MalkoskiSP, WangXJ. Two sides of the story? FEBS Lett 2012;586:1984–92. DOI PubMed PMC22321641 10.1016/j.febslet.2012.01.054PMC3285395

[44] ZhaoM, MishraL, DengCX. The role of TGF-β/SMAD4 signaling in cancer. Int J Biol Sci 2018;14:111–23. DOI PubMed PMC29483830 10.7150/ijbs.23230PMC5821033

[45] LecandaJ, GanapathyV, D’Aquino-ArdalanC, TGFbeta prevents proteasomal degradation of the cyclin-dependent kinase inhibitor p27kip1 for cell cycle arrest. Cell Cycle 2009;8:742–56. DOI PubMed19221482 10.4161/cc.8.5.7871

[46] AdamskaA, ElaskalaniO, EmmanouilidiA, Molecular and cellular mechanisms of chemoresistance in pancreatic cancer. Adv Biol Regul 2018;68:77–87. DOI PubMed29221990 10.1016/j.jbior.2017.11.007

[47] MackeyJR, ManiRS, SelnerM, Functional nucleoside transporters are required for gemcitabine influx and manifestation of toxicity in cancer cell lines. Cancer Res 1998;58:4349–57. PubMed9766663

[48] Sousa CavalcanteL, MonteiroG. Gemcitabine: metabolism and molecular mechanisms of action, sensitivity and chemoresistance in pancreatic cancer. Eur J Pharmacol 2014;741:8–16. DOI PubMed25084222 10.1016/j.ejphar.2014.07.041

[49] BinenbaumY, Na’araS, GilZ. Gemcitabine resistance in pancreatic ductal adenocarcinoma. Drug Resist Updat 2015;23:55–68. DOI PubMed26690340 10.1016/j.drup.2015.10.002

[50] HungSW, ModyHR, GovindarajanR. Overcoming nucleoside analog chemoresistance of pancreatic cancer: a therapeutic challenge. Cancer Lett 2012;320:138–49. DOI PubMed PMC22425961 10.1016/j.canlet.2012.03.007PMC3569094

[51] SkrypekN, DuchêneB, HebbarM, LeteurtreE, van SeuningenI, JonckheereN. The MUC4 mucin mediates gemcitabine resistance of human pancreatic cancer cells via the Concentrative Nucleoside Transporter family. Oncogene 2013;32:1714–23. DOI PubMed PMC22580602 10.1038/onc.2012.179PMC3936121

[52] SchinkelAH, JonkerJW. Mammalian drug efflux transporters of the ATP binding cassette (ABC) family: an overview. Adv Drug Deliv Rev 2003;55:3–29. DOI PubMed12535572 10.1016/s0169-409x(02)00169-2

[53] MaréchalR, MackeyJR, LaiR, Deoxycitidine kinase is associated with prolonged survival after adjuvant gemcitabine for resected pancreatic adenocarcinoma. Cancer 2010;116:5200–6. DOI PubMed20669326 10.1002/cncr.25303

[54] BergmanAM, EijkPP, Ruiz van HaperenVW, In vivo induction of resistance to gemcitabine results in increased expression of ribonucleotide reductase subunit M1 as the major determinant. Cancer Res 2005;65:9510–6. DOI PubMed16230416 10.1158/0008-5472.CAN-05-0989

[55] DavidsonJD, MaL, FlagellaM, GeeganageS, GelbertLM, SlapakCA. An increase in the expression of ribonucleotide reductase large subunit 1 is associated with gemcitabine resistance in non-small cell lung cancer cell lines. Cancer Res 2004;64:3761–6. DOI PubMed15172981 10.1158/0008-5472.CAN-03-3363

[56] AsanoT, YaoY, ZhuJ, LiD, AbbruzzeseJL, ReddySA. The PI 3-kinase/Akt signaling pathway is activated due to aberrant Pten expression and targets transcription factors NF-kappaB and c-Myc in pancreatic cancer cells. Oncogene 2004;23:8571–80. DOI PubMed15467756 10.1038/sj.onc.1207902

[57] SinghK Therapeutic targeting of deregulated cell cycle and UPR in pancreatic cancer by tetrandrine. PhD, Biochemistry and Molecular Biology, Louisiana State University Health Sciences Center at Shreveport, ProQuest, 2018.

[58] EbrahimiS, HosseiniM, ShahidsalesS, Targeting the Akt/PI3K signaling pathway as a potential therapeutic strategy for the treatment of pancreatic cancer. Curr Med Chem 2017;24:1321–31. DOI PubMed28176634 10.2174/0929867324666170206142658

[59] YamamotoS, TomitaY, HoshidaY, Prognostic significance of activated Akt expression in pancreatic ductal adenocarcinoma. Clin Cancer Res 2004;10:2846–50. DOI PubMed15102693 10.1158/1078-0432.ccr-02-1441

[60] ParsonsCM, MuilenburgD, BowlesTL, VirudachalamS, BoldRJ. The role of Akt activation in the response to chemotherapy in pancreatic cancer. Anticancer Res 2010;30:3279–89. PubMed PMC20944098 PMC4557882

[61] NgSSW, TsaoMS, ChowS, HedleyDW. Inhibition of phosphatidylinositide 3-kinase enhances gemcitabine-induced apoptosis in human pancreatic cancer cells. Cancer Res 2000;60:5451–5. PubMed11034087

[62] XieD, XieK. Pancreatic cancer stromal biology and therapy. Genes Dis 2015;2:133–43. DOI PubMed PMC26114155 10.1016/j.gendis.2015.01.002PMC4476547

[63] WaghrayM, YalamanchiliM, di MaglianoMP, SimeoneDM. Deciphering the role of stroma in pancreatic cancer. Curr Opin Gastroenterol 2013;29:537–43. DOI PubMed PMC23892539 10.1097/MOG.0b013e328363affePMC4112589

[64] NielsenMF, MortensenMB, DetlefsenS. Key players in pancreatic cancer-stroma interaction: Cancer-associated fibroblasts, endothelial and inflammatory cells. World J Gastroenterol 2016;22:2678–700. DOI PubMed PMC26973408 10.3748/wjg.v22.i9.2678PMC4777992

[65] WalterK, OmuraN, HongSM, Overexpression of smoothened activates the sonic hedgehog signaling pathway in pancreatic cancer-associated fibroblasts. Clin Cancer Res 2010;16:1781–9. DOI PubMed PMC20215540 10.1158/1078-0432.CCR-09-1913PMC2846609

[66] GoreJ, KorcM. Pancreatic cancer stroma: friend or foe? Cancer Cell 2014;25:711–2. DOI PubMed PMC24937454 10.1016/j.ccr.2014.05.026PMC4821630

[67] LiH, ZhangJ, ChenSW, Cancer-associated fibroblasts provide a suitable microenvironment for tumor development and progression in oral tongue squamous cancer. J Transl Med 2015;13:198. DOI PubMed PMC26094024 10.1186/s12967-015-0551-8PMC4475624

[68] HeldinCH, RubinK, PietrasK, OstmanA. High interstitial fluid pressure - an obstacle in cancer therapy. Nat Rev Cancer 2004;4:806–13. DOI PubMed15510161 10.1038/nrc1456

[69] LiangC, ShiS, MengQ, Complex roles of the stroma in the intrinsic resistance to gemcitabine in pancreatic cancer: where we are and where we are going. Exp Mol Med 2017;49:e406. DOI PubMed PMC29611542 10.1038/emm.2017.255PMC5750480

[70] KozonoS, OhuchidaK, EguchiD, Pirfenidone inhibits pancreatic cancer desmoplasia by regulating stellate cells. Cancer Res 2013;73:2345–56. DOI PubMed23348422 10.1158/0008-5472.CAN-12-3180

[71] JacobetzMA, ChanDS, NeesseA, Hyaluronan impairs vascular function and drug delivery in a mouse model of pancreatic cancer. Gut 2013;62:112–20. DOI PubMed PMC22466618 10.1136/gutjnl-2012-302529PMC3551211

[72] ProvenzanoPP, CuevasC, ChangAE, GoelVK, Von HoffDD, HingoraniSR. Enzymatic targeting of the stroma ablates physical barriers to treatment of pancreatic ductal adenocarcinoma. Cancer Cell 2012;21:418–29. DOI PubMed PMC22439937 10.1016/j.ccr.2012.01.007PMC3371414

[73] HingoraniSR, HarrisWP, BeckJT, Phase Ib study of PEGylated recombinant human hyaluronidase and gemcitabine in patients with advanced pancreatic cancer. Clin Cancer Res 2016;22:2848–54. DOI PubMed PMC26813359 10.1158/1078-0432.CCR-15-2010PMC7787348

[74] AlvarezR, MusteanuM, Garcia-GarciaE, Stromal disrupting effects of nab-paclitaxel in pancreatic cancer. Br J Cancer 2013;109:926–33. DOI PubMed PMC23907428 10.1038/bjc.2013.415PMC3749580

[75] Von HoffDD, ErvinT, ArenaFP, Increased survival in pancreatic cancer with nab-paclitaxel plus gemcitabine. N Engl J Med 2013;369:1691–703. DOI PubMed PMC24131140 10.1056/NEJMoa1304369PMC4631139

[76] HotzHG, ReberHA, HotzB, Angiogenesis inhibitor TNP-470 reduces human pancreatic cancer growth. J Gastrointest Surg 2001;5:131–8. DOI PubMed11331474 10.1016/s1091-255x(01)80024-x

[77] OliveKP, JacobetzMA, DavidsonCJ, Inhibition of Hedgehog signaling enhances delivery of chemotherapy in a mouse model of pancreatic cancer. Science 2009;324:1457–61. DOI PubMed PMC19460966 10.1126/science.1171362PMC2998180

[78] OlivaresO, MayersJR, GouirandV, Collagen-derived proline promotes pancreatic ductal adenocarcinoma cell survival under nutrient limited conditions. Nat Commun 2017;8:16031. DOI PubMed PMC28685754 10.1038/ncomms16031PMC5504351

[79] HoseinAN, BrekkenRA, MaitraA. Pancreatic cancer stroma: an update on therapeutic targeting strategies. Nat Rev Gastroenterol Hepatol 2020;17:487–505. DOI PubMed PMC32393771 10.1038/s41575-020-0300-1PMC8284850

[80] VenninC, ChinVT, WarrenSC, ; Australian Pancreatic Cancer Genome Initiative (APGI). Transient tissue priming via ROCK inhibition uncouples pancreatic cancer progression, sensitivity to chemotherapy, and metastasis. Sci Transl Med 2017;9:eaai8504. DOI PubMed PMC10.1126/scitranslmed.aai8504PMC577750428381539

[81] JiangH, HegdeS, KnolhoffBL, Targeting focal adhesion kinase renders pancreatic cancers responsive to checkpoint immunotherapy. Nat Med 2016;22:851–60. DOI PubMed PMC27376576 10.1038/nm.4123PMC4935930

[82] AguileraKY, HuangH, DuW, Inhibition of discoidin domain receptor 1 reduces collagen-mediated tumorigenicity in pancreatic ductal adenocarcinoma. Mol Cancer Ther 2017;16:2473–85. DOI PubMed PMC28864681 10.1158/1535-7163.MCT-16-0834PMC5669827

[83] BoucherMJ, MorissetJ, VachonPH, ReedJC, LainéJ, RivardN. MEK/ERK signaling pathway regulates the expression of Bcl-2, Bcl-X(L), and Mcl-1 and promotes survival of human pancreatic cancer cells. J Cell Biochem 2000;79:355–69. PubMed10972974

[84] WangM, LuX, DongX, pERK1/2 silencing sensitizes pancreatic cancer BXPC-3 cell to gemcitabine-induced apoptosis via regulating Bax and Bcl-2 expression. World J Surg Oncol 2015;13:66. DOI PubMed PMC25880226 10.1186/s12957-015-0451-7PMC4337256

[85] KarimianA, AhmadiY, YousefiB. Multiple functions of p21 in cell cycle, apoptosis and transcriptional regulation after DNA damage. DNA Repair (Amst) 2016;42:63–71. DOI PubMed27156098 10.1016/j.dnarep.2016.04.008

[86] ChienW, DingLW, SunQY, Selective inhibition of unfolded protein response induces apoptosis in pancreatic cancer cells. Oncotarget 2014;5:4881–94. DOI PubMed PMC24952679 10.18632/oncotarget.2051PMC4148107

[87] MaY, HendershotLM. The role of the unfolded protein response in tumour development: friend or foe? Nat Rev Cancer 2004;4:966–77. DOI PubMed15573118 10.1038/nrc1505

[88] YousefiH, YuanJ, Keshavarz-FathiM, MurphyJF, RezaeiN. Immunotherapy of cancers comes of age. Expert Rev Clin Immunol 2017;13:1001–15. DOI PubMed28795649 10.1080/1744666X.2017.1366315

[89] PatelSP, KurzrockR. PD-L1 expression as a predictive biomarker in cancer immunotherapy. Mol Cancer Ther 2015;14:847–56. DOI PubMed25695955 10.1158/1535-7163.MCT-14-0983

[90] BeattyGL, ChioreanEG, FishmanMP, CD40 agonists alter tumor stroma and show efficacy against pancreatic carcinoma in mice and humans. Science 2011;331:1612–6. DOI PubMed PMC21436454 10.1126/science.1198443PMC3406187

[91] De MonteL, ReniM, TassiE, Intratumor T helper type 2 cell infiltrate correlates with cancer-associated fibroblast thymic stromal lymphopoietin production and reduced survival in pancreatic cancer. J Exp Med 2011;208:469–78. DOI PubMed PMC21339327 10.1084/jem.20101876PMC3058573

[92] FukunagaA, MiyamotoM, ChoY, CD8+ tumor-infiltrating lymphocytes together with CD4+ tumor-infiltrating lymphocytes and dendritic cells improve the prognosis of patients with pancreatic adenocarcinoma. Pancreas 2004;28:e26–31. DOI PubMed14707745 10.1097/00006676-200401000-00023

[93] SchizasD, CharalampakisN, KoleC, Immunotherapy for pancreatic cancer: a 2020 update. Cancer Treat Rev 2020;86:102016. DOI PubMed32247999 10.1016/j.ctrv.2020.102016

[94] RoyalRE, LevyC, TurnerK, Phase 2 trial of single agent Ipilimumab (anti-CTLA-4) for locally advanced or metastatic pancreatic adenocarcinoma. J Immunother 2010;33:828–33. DOI PubMed PMC20842054 10.1097/CJI.0b013e3181eec14cPMC7322622

[95] KalyanA, KircherSM, MohindraNA, Ipilimumab and gemcitabine for advanced pancreas cancer: a phase Ib study. J Clin Oncol 2016;34:e15747. DOI10.1634/theoncologist.2019-0473PMC721643631740568

[96] LuoW, YangG, LuoW, Novel therapeutic strategies and perspectives for metastatic pancreatic cancer: vaccine therapy is more than just a theory. Cancer Cell Int 2020;20:66. DOI PubMed PMC32158356 10.1186/s12935-020-1147-9PMC7057654

[97] MiyazawaM, KatsudaM, MaguchiH, Phase II clinical trial using novel peptide cocktail vaccine as a postoperative adjuvant treatment for surgically resected pancreatic cancer patients. Int J Cancer 2017;140:973–82. DOI PubMed27861852 10.1002/ijc.30510

[98] MuccioloG, RouxC, ScagliottiA, BrugiapagliaS, NovelliF, CappelloP. The dark side of immunotherapy: pancreatic cancer. Cancer Drug Resist 2020;3:491–520. DOI35582441 10.20517/cdr.2020.13PMC8992483

[99] LeDT, LutzE, UramJN, Evaluation of ipilimumab in combination with allogeneic pancreatic tumor cells transfected with a GM-CSF gene in previously treated pancreatic cancer. J Immunother 2013;36:382–9. DOI PubMed PMC23924790 10.1097/CJI.0b013e31829fb7a2PMC3779664

[100] HolterS, BorgidaA, DoddA, Germline BRCA mutations in a large clinic-based cohort of patients with pancreatic adenocarcinoma. J Clin Oncol 2015;33:3124–9. DOI PubMed25940717 10.1200/JCO.2014.59.7401

[101] Salo-MullenEE, O’ReillyEM, KelsenDP, Identification of germline genetic mutations in patients with pancreatic cancer. Cancer 2015;121:4382–8. DOI PubMed PMC26440929 10.1002/cncr.29664PMC5193099

[102] WaddellN, PajicM, PatchAM, ; Australian Pancreatic Cancer Genome Initiative. Whole genomes redefine the mutational landscape of pancreatic cancer. Nature 2015;518:495–501. DOI PubMed PMC25719666 10.1038/nature14169PMC4523082

[103] LordCJ, AshworthA. BRCAness revisited. Nat Rev Cancer 2016;16:110–20. DOI PubMed26775620 10.1038/nrc.2015.21

[104] GuptaM, IyerR, FountzilasC. Poly(ADP-Ribose) Polymerase inhibitors in pancreatic cancer: a new treatment paradigms and future implications. Cancers (Basel) 2019;11:1980. DOI PubMed PMC31835379 10.3390/cancers11121980PMC6966572

[105] GolanT, HammelP, ReniM, Maintenance olaparib for germline BRCA-mutated metastatic pancreatic cancer. N Engl J Med 2019;381:317–27. DOI PubMed PMC31157963 10.1056/NEJMoa1903387PMC6810605

[106] ZhuH, WeiM, XuJ, PARP inhibitors in pancreatic cancer: molecular mechanisms and clinical applications. Mol Cancer 2020;19:49. DOI PubMed PMC32122376 10.1186/s12943-020-01167-9PMC7053129

[107] LerouxC, KonstantinidouG. Targeted therapies for pancreatic cancer: overview of current treatments and new opportunities for personalized oncology. Cancers (Basel) 2021;13:799. DOI PubMed PMC33672917 10.3390/cancers13040799PMC7918504

[108] LawrenceTS, ChangEY, HahnTM, HertelLW, ShewachD. Radiosensitization of pancreatic cancer cells by 2′,2′-difluoro-2′deoxycytidine. International Journal of Radiation Oncology*Biology*Physics 1996;34:867–72. DOI PubMed8598364 10.1016/0360-3016(95)02134-5

[109] DoyleTH, MornexF, McKennaWG. The clinical implications of gemcitabine radiosensitization. Clin Cancer Res 2001;7:226–8. PubMed11234872

[110] TuliR, SurmakAJ, ReyesJ, Radiosensitization of pancreatic cancer cells in vitro and in vivo through poly (ADP-ribose) polymerase inhibition with ABT-888. Transl Oncol ;2014:439–45. DOI PubMed PMC24836647 10.1016/j.tranon.2014.04.003PMC4145354

[111] JiangY, DaiH, LiY, PARP inhibitors synergize with gemcitabine by potentiating DNA damage in non-small-cell lung cancer. Int J Cancer 2019;144:1092–103. DOI PubMed PMC30152517 10.1002/ijc.31770PMC6320711

[112] WaissiW, NicolA, JungM, Radiosensitizing pancreatic cancer with PARP inhibitor and gemcitabine: an in vivo and a wholetranscriptome analysis after proton or photon irradiation. Cancers (Basel) 2021;13:527. DOI PubMed PMC33573176 10.3390/cancers13030527PMC7866541

[113] ÖhlundD, ElyadaE, TuvesonD. Fibroblast heterogeneity in the cancer wound. J Exp Med 2014;211:1503–23. DOI PubMed PMC25071162 10.1084/jem.20140692PMC4113948

[114] FioriME, Di FrancoS, VillanovaL, BiancaP, StassiG, De MariaR. Cancer-associated fibroblasts as abettors of tumor progression at the crossroads of EMT and therapy resistance. Mol Cancer 2019;18:70. DOI PubMed PMC30927908 10.1186/s12943-019-0994-2PMC6441236

[115] DomenA, QuatannensD, ZanivanS, Cancer-associated fibroblasts as a common orchestrator of therapy resistance in lung and pancreatic cancer. Cancers (Basel) 2021;13:987. DOI PubMed PMC33673405 10.3390/cancers13050987PMC7956441

[116] WuQ, TianY, ZhangJ, Functions of pancreatic stellate cell-derived soluble factors in the microenvironment of pancreatic ductal carcinoma. Oncotarget 2017;8:102721–38. DOI PubMed PMC29254283 10.18632/oncotarget.21970PMC5731993

[117] Ene-ObongA, ClearAJ, WattJ, Activated pancreatic stellate cells sequester CD8+ T cells to reduce their infiltration of the juxtatumoral compartment of pancreatic ductal adenocarcinoma. Gastroenterology 2013;145:1121–32. DOI PubMed PMC23891972 10.1053/j.gastro.2013.07.025PMC3896919

[118] KumarV, DonthireddyL, MarvelD, Cancer-associated fibroblasts neutralize the anti-tumor effect of CSF1 receptor blockade by inducing PMN-MDSC infiltration of tumors. Cancer Cell 2017;32:654–68.e5. DOI PubMed PMC29136508 10.1016/j.ccell.2017.10.005PMC5827952

[119] FrancesconeR, Barbosa Vendramini-CostaD, Franco-BarrazaJ, Netrin G1 promotes pancreatic tumorigenesis through cancer-associated fibroblast-driven nutritional support and immunosuppression. Cancer Discov 2021;11:446–79. DOI PubMed PMC33127842 10.1158/2159-8290.CD-20-0775PMC7858242

[120] CohenSJ, AlpaughRK, PalazzoI, Fibroblast activation protein and its relationship to clinical outcome in pancreatic adenocarcinoma. Pancreas 2008;37:154–8. DOI PubMed18665076 10.1097/MPA.0b013e31816618ce

[121] SunamiY, BökerV, KleeffJ. Targeting and reprograming cancer-associated fibroblasts and the tumor microenvironment in pancreatic cancer. Cancers (Basel) 2021;13:697. DOI PubMed PMC33572223 10.3390/cancers13040697PMC7915918

[122] ZhangY, RecouvreuxMV, JungM, Macropinocytosis in cancer-associated fibroblasts is dependent on CaMKK2/ARHGEF2 signaling and functions to support tumor and stromal cell fitness. Cancer Discov 2021;11:1808–25. DOI PubMed PMC33653692 10.1158/2159-8290.CD-20-0119PMC8292164

[123] DavidsonSM, JonasO, KeiblerMA, Direct evidence for cancer-cell-autonomous extracellular protein catabolism in pancreatic tumors. Nat Med 2017;23:235–41. DOI PubMed PMC28024083 10.1038/nm.4256PMC5407288

[124] NeoptolemosJP, KleeffJ, MichlP, CostelloE, GreenhalfW, PalmerDH. Therapeutic developments in pancreatic cancer: current and future perspectives. Nat Rev Gastroenterol Hepatol 2018;15:333–48. DOI PubMed29717230 10.1038/s41575-018-0005-x

[125] ConroyT, DesseigneF, YchouM, ; Groupe Tumeurs Digestives of Unicancer; PRODIGE Intergroup. FOLFIRINOX versus gemcitabine for metastatic pancreatic cancer. N Engl J Med 2011;364:1817–25. DOI PubMed21561347 10.1056/NEJMoa1011923

